# Whole-genome sequencing suggests mechanisms for 22q11.2 deletion-associated Parkinson’s disease

**DOI:** 10.1371/journal.pone.0173944

**Published:** 2017-04-21

**Authors:** Nancy J. Butcher, Daniele Merico, Mehdi Zarrei, Lucas Ogura, Christian R. Marshall, Eva W. C. Chow, Anthony E. Lang, Stephen W. Scherer, Anne S. Bassett

**Affiliations:** 1 Clinical Genetics Research Program, Centre for Addiction and Mental Health, Toronto, Ontario, Canada; 2 Campbell Family Mental Health Research Institute, Centre for Addiction and Mental Health, Toronto, Ontario, Canada; 3 Institute of Medical Science, University of Toronto, Toronto, Ontario, Canada; 4 The Centre for Applied Genomics and Program in Genetics and Genome Biology, The Hospital for Sick Children, Toronto, Ontario, Canada; 5 McLaughlin Centre and Department of Molecular Genetics, University of Toronto, Toronto, Ontario, Canada; 6 Department of Psychiatry, University of Toronto, Toronto, Ontario, Canada; 7 Division of Neurology, Department of Medicine, University of Toronto, Toronto, Ontario, Canada; 8 Tanz Centre for Research in Neurodegenerative Diseases, Department of Medicine, University of Toronto, Toronto, Ontario, Canada; 9 Toronto Western Hospital Research Institute, University Health Network, Toronto, Ontario, Canada; 10 Edmond J. Safra Program in Parkinson’s Disease, Toronto Western Hospital, Toronto, Ontario, Canada; 11 Toronto General Research Institute, University Health Network, Toronto, Ontario, Canada; 12 The Dalglish 22q Clinic for Adults with 22q11.2 Deletion Syndrome, University Health Network, Toronto, Ontario, Canada; 13 Department of Psychiatry, University Health Network, Toronto, Ontario, Canada; 14 Division of Cardiology, Department of Medicine, University Health Network, Toronto, Ontario, Canada; CNR, ITALY

## Abstract

**Objectives:**

To investigate disease risk mechanisms of early-onset Parkinson’s disease (PD) associated with the recurrent 22q11.2 deletion, a genetic risk factor for early-onset PD.

**Methods:**

In a proof-of-principle study, we used whole-genome sequencing (WGS) to investigate sequence variants in nine adults with 22q11.2DS, three with neuropathologically confirmed early-onset PD and six without PD. Adopting an approach used recently to study schizophrenia in 22q11.2DS, here we tested candidate gene-sets relevant to PD.

**Results:**

No mutations common to the cases with PD were found in the intact 22q11.2 region. While all were negative for rare mutations in a gene-set comprising PD disease-causing and risk genes, another candidate gene-set of 1000 genes functionally relevant to PD presented a nominally significant (*P* = 0.03) enrichment of rare putatively damaging missense variants in the PD cases. Polygenic score results, based on common variants associated with PD risk, were non-significantly greater in those with PD.

**Conclusions:**

The results of this first-ever pilot study of WGS in PD suggest that the cumulative burden of genome-wide sequence variants may contribute to expression of early-onset PD in the presence of threshold-lowering dosage effects of a 22q11.2 deletion. We found no evidence that expression of PD in 22q11.2DS is mediated by a recessive locus on the intact 22q11.2 chromosome or mutations in known PD genes. These findings offer initial evidence of the potential effects of multiple within-individual rare variants on the expression of PD and the utility of next generation sequencing for studying the etiology of PD.

## Introduction

Over the past two decades new knowledge has elucidated a genetic basis for an increasing proportion of patients with Parkinson’s disease (PD) [1]. We identified the hemizygous 22q11.2 microdeletion associated with 22q11.2 deletion syndrome (22q11.2DS; OMIM #192430, #188400) as a novel genetic risk factor for neuropathologically confirmed, L-dopa responsive early-onset PD [2]. This structural variant has since been found to be significantly enriched in early-onset PD cohorts and may account for ~0.5% of cases [3]. Genetic variants on the intact 22q11.2 chromosome, or genome-wide outside of the deletion region, may influence the likelihood of expression of PD in 22q11.2DS. Using this genetic model to identify such variants could provide clues to variants that affect susceptibility to idiopathic forms of PD and general disease mechanisms.

As a proof-of-principle to assess this model, we used whole-genome sequencing (WGS) data, adapting a strategy successfully used to study expression of schizophrenia in 22q11.2DS [4]. We compared variants from 22q11.2DS patients with early-onset PD [2] to those without PD. To maximize statistical power in this initial study, we investigated rare variant burden for gene-sets with higher a priori likelihood of contributing to PD risk. These included variants affecting candidate genes in the 22q11.2 deletion region, such as *COMT*, *SEPT5*, and six mitochondrial function genes, as well as other genome-wide PD-relevant gene-sets. We also investigated common variant contribution using a polygenic risk score model. We found evidence for rare variants outside the 22q11.2 region perturbing gene networks relevant to PD, supporting the utility of this genetic model for early-onset PD.

## Methods

### Subjects

We performed WGS using DNA from nine unrelated adults of European descent with chromosome 22q11.2 deletions [4] (Table 1) selected from a cohort of Canadian adults with 22q11.2DS [2, 5–8]. Direct clinical assessments at multiple timepoints and review of comprehensive lifetime medical records and clinical histories provided deep phenotyping using our established methods [2, 4–8]. The three subjects with early-onset PD (PD1-PD3) met United Kingdom Parkinson’s Disease Society Brain Bank clinical diagnostic criteria and had confirmed PD neuropathology [2]. We had previously reported that these 22q11.2DS-PD patients had no known pathogenic point mutations in *LRRK2*, *PARK2*, *DJ-I*, *PINK1*, or *SNCA*, or copy number variants in *PARK2* or *SNCA* [2].

**Table 1:** Clinical and 22q11.2 deletion-related characteristics of individuals with 22q11.2 deletion syndrome of European Ancestry with whole-genome sequencing results.

	22q11.2DS-Parkinson’s disease			22q11.2DS-No Parkinson’s disease					
*Subject identifier*	PD1	PD2	PD3	NPD1	NPD2	NPD3	NPD4	NPD5	NPD6
***Demographic features and PD phenotype***									
Sex	Female	Male	Male	Female	Male	Female	Male	Male	Female
Age (y)									
At last follow-up or at death	56	58	61	21	38	48	44	53	52
PD motor symptom onset	45	48	43	-	-	-	-	-	-
PD diagnosis	55	54	44	-	-	-	-	-	-
Family history of PD	No	No	No	No	No	No	No	Father, late-onset	No
Neuropathology^a^	Nigral cell death, LBs [2]	Nigral cell death, LBs [2]	Nigral cell death [2]	Living	Living	Living	No PD pathology [10]	Living	Living
***Other 22q11*.*2DS features***									
Congenital heart defect	No	Yes^b^	No	Yes^b^	No	No	Yes^b^	No	Yes^b^
Intellectual disability	Borderline	None	None	Mild	Mild	Borderline	Borderline	Borderline	Borderline
Schizophrenia (age at onset, y)	Yes (17)	Yes (22)	No	Yes (12)	Yes (15)	Yes (18)	Yes (21)	No	No
Other psychiatric disorder^c^	No	No	No	Yes	No	No	No	Yes	Yes
Seizures^d^	Single	No	No	Recurrent	Recurrent	Recurrent	Single	No	No
***22q11*.*2 deletion region***									
Deletion type^e^	Nested–proximal	Typical	Typical	Typical	Typical	Typical	Typical	Typical	Typical
*De novo* 22q11.2 deletion	Probable	Probable	Probable	Probable	Yes	Yes	Probable	Yes	Probable

Abbreviations: 22q11.2DS, 22q11.2 deletion syndrome; LBs, Lewy bodies; NPD, No Parkinson’s disease; PD, Parkinson’s disease; VPI, velopharyngeal insufficiency; y, year

^a^Detailed phenotypic reports published previously for PD1-PD3 [2] and NPD4 [10]

^b^Tetralogy of Fallot (PD2); ventricular septal defect (NPD1); ventricular septal defect and atrial septal defect (NPD4, NPD6)

^c^Lifetime history; Other psychiatric disorders were obsessive compulsive disorder (NPD1), generalized anxiety disorder (NPD5, NPD6)

^d^Lifetime history; Note all subjects had a lifetime history of hypocalcemia

^e^Age at molecular diagnosis of 22q11.2 deletion was 44, 52, 53 years for subjects with Parkinson’s disease and 4, 21, 27, 39, 41, 42 years for subjects without Parkinson’s disease

Informed consent was obtained in writing and the study was approved by research ethics boards at the Centre for Addiction and Mental Health, Toronto, Ontario, Canada.

### Whole-genome sequencing

The overall WGS approach was based on methods used in recent studies of schizophrenia [4] and autism [9]. Detailed laboratory and bioinformatics methods are described elsewhere [4]. In brief, genomic DNA was extracted from whole blood and sequenced using the Complete Genomics platform (pipeline and assembly version 2.2). On average, 99.0% of the genome was covered with at least 5x sequence depth relative to the NCBI build 37 human genome reference sequence. Of the exome, 94.4% and 72.3% was covered with at least 20x and 40x sequence depth, respectively. Only high-quality variants, defined as those with Complete Genomics “highquality” variant call scores and that also met stringent in-house quality criteria [4, 9] were included. The effects (e.g., nonsense, missense, or frameshift mutations) and classifications (e.g., in exonic, intronic, or intergenic regions) of variants across the genome were annotated using ANNOVAR (November 2014) software [11]. Rarity was annotated using the three major publicly-available reference datasets based on WGS and whole exome sequencing, i.e., 1000 Genomes [12], NHLBI-Exome Sequencing Project [13] and Exome Aggregation Consortium [14], and two in-house platform-matched whole-genome reference data-sets. We defined rare variants using a standard rarity threshold of those not exceeding the 1% alternate allele frequency threshold (minor allele frequency [MAF]<0.01) in all datasets, including specific ethnic subgroups.

### Non-synonymous and structural variants

We examined rare single-nucleotide sequence variants (SNVs) and in/dels affecting coding genes that included loss-of-function (stop-gain/nonsense, frameshift, and core splice site alterations) and missense mutations categorized as deleterious using standard variant impact predictor tools and genomic conservation indexes, as reported previously [4, 9]. Missense variants were predicted damaging (deleterious) when they met at least four of seven criteria: high conservation by PhyloP placental mammal (≥2.3) and PhyloP 100vertebrate (≥4) [15] and predicted damaging by SIFT ≤0.05 [16], PolyPhen2 ≥0.90 [17], Mutation Assessor ≥1.9 [18], CADD Phred score ≥15 [19], and MutationTaster score >0.5 [20]. We restricted the analyses to diploid regions of the genome with the exception of the 22q11.2 deletion to help manage the risk of false positives.

Copy number variant and other structural variants detected by Complete Genomics pipeline and assembly version 2.2 were annotated and filtered as described elsewhere [4, 9]. We considered only high-quality rare variants that overlapped a coding gene exon of a RefSeq gene [4, 9]. 22q11.2 deletions were confirmed in all patients (Table 1).

### Gene-set analyses

The analysis was restricted to a candidate gene approach of rare variants involving coding regions of the genome. Rare coding sequence variants are enriched for those that are deleterious and have a moderate to large effect on disease risk. We have previously shown that this approach, even for the small sample selected for our pilot WGS study of 22q11.2DS, can yield substantial effect sizes for a neuropsychiatric phenotype [4]. Three hypothesis-driven gene-sets were selected *a priori* for testing based on proposed possible PD mechanisms in 22q11.2DS [2, 21]: (1) 22q11.2 deletion region genes (n = 46) [22]; (2) known causative and PD risk candidate genes (n = 43); and (3) genes identified as functionally-relevant to PD (n = 1000) based on physical protein interactions and co-expression with known PD gene candidates, excluding the 22q11.2 region and the known causative and risk PD genes that were examined as separate gene-sets.

The known candidate PD gene-set (S1 Table) included genes described in the scientific literature and/or, OMIM entries for Parkinson’s disease (#168600), and from the PDgene database of genome-wide association study common variant findings (www.pdgene.org; accessed October 2015) [23, 24]. We used this approach [25] because rare and common variants can involve the same PD risk gene (e.g., *SNCA*) [1, 23, 24]. We opted to assess all genes (e.g., confirmed, unconfirmed, and rarely) reported to be involved in Parkinson’s disease in order to limit the possibility of a false negative with respect to susceptibility to Parkinson’s disease in patients with 22q11.2DS.

The gene-set of 1000 “PD-relevant” genes was generated using the genome-wide candidate gene prioritization tool, Endeavour [26]. Endeavour prioritizes genes by assessing how similar they are to genes already known to be involved in the process of interest [26–28]. To generate a genome-wide ranked list of candidate “PD-relevant” genes, the 43 genes comprising the known PD gene-set (S1 Table) were used as input training genes with data imported from standard annotation databases (Gene Ontology, Kegg, SwissProt), protein-protein interaction databases (BIND, BioGRID, Hprd, InNetDb, Intact, Mint), and a human gene expression database [29]. This yielded 1561 ranked genes mapped to current unique HGNC gene symbols (S2 Table). We *a priori* restricted the PD-relevant gene-set to the highest-ranked 1000 genes (“top 1000 genes”; S2 Table) to help prevent the inclusion of genes with weak evidence for involvement in PD-related function, while including a sufficient number of genes to perform a proof-of-principle genome-wide burden analysis [4]. As a negative control, we generated 10 000 random gene-sets composed of 1000 “non-PD relevant” genes each from a set of 14 489 genes that met the following criteria: (1) at least one Gene Ontology [30] annotation with ≤ 500 genes, to ensure that the genes were annotated with a functional term that was sufficiently specific, and (2) were not in the known candidate PD gene-set or the PD-relevant gene-set (top 1000).

### Common variant polygenic risk score

We calculated the polygenic score using the approach previously described for 22q11.2DS-associated schizophrenia [4] and the International Schizophrenia Consortium [31]. Our analysis was restricted to the top 10 000 most significant SNPs with original nominal association *p*-values and odds-ratios that are publicly available from PDgene [23, 24]. Of these, 3534 SNPs were mapped to variants passing quality filters in all nine genomes assessed in this study. Allele counts were computed as the number of alleles that matched to the allele used for association analysis. The SNP-wise risk score was calculated as the product of the allele count and the log (odds-ratio). The polygenic risk score [4] for each 22q11.2DS subject was calculated as the sum of all respective SNP-wise risk scores using nominal association *p*-value thresholds ≤1e^-3^ and ≤1e^-5^, as well as one more stringent (≤1e^-7^) corresponding to 3410, 1315, and 388 SNPs each.

### Statistical analyses

We used one-sided independent *t-*tests in these exploratory analyses to assess rare variant burden count and common variant polygenic risk score between groups, performed with SAS version 9.4 software. Statistical significance was defined using nominal uncorrected *p*<0.05. The false discovery rate (FDR) was calculated using the Benjamini-Hochberg procedure.

## Results

Table 1 summarizes the demographic, clinical, and 22q11.2 deletion data for the subjects studied. No feature of 22q11.2DS, apart from the presence of neuropathologically-confirmed PD, was unique to the 22q11.2DS-PD cases. The clinical heterogeneity among the subjects was typical for 22q11.2DS [32–34]. Expression of schizophrenia was similarly common (66.6%) in both groups. Among the subjects with PD, there was a history of borderline intellectual disability and an isolated seizure in one subject and a congenital heart defect in another. Major co-morbid conditions among the patients without PD included intellectual disability (n = 2 mild; n = 4 borderline), seizures (n = 3 recurrent; n = 1 single), congenital heart defects (n = 3), and other psychiatric disorders including obsessive compulsive disorder (n = 1) and generalized anxiety disorder (n = 2). Patients in the PD group were diagnosed with 22q11.2DS at an older mean age (49.6±4.9 years) than those without PD (29±14.9 years, *p* = 0.048). One subject with PD had a 1.4 Mb nested proximal 22q11.2 deletion (S3 Table). The deletion breakpoints were consistent with typical deletions in the other eight subjects (n = 7, 2.6 Mb deletion including two with PD; n = 1, 2.9 Mb deletion; S3 Table). There were no deleterious nonsynonymous variants near the 22q11.2 deletion breakpoints (i.e., within 4 Mb).

WGS results for the 22q11.2 deletion region revealed only a single rare missense variant involving different brain-expressed genes in each of three subjects (Table 2). None involved a putative 22q11.2 region PD candidate gene [2, 21] and the variants in cases PD1 and PD3 involved genes *TRMT2A* and *DGCR2*, encoding proteins of largely unknown function (Table 2) [35, 36].

**Table 2:** Rare nonsynonymous variants in 22q11.2 deletion-associated early-onset Parkinson’s disease (22q11.2ds-pd) patients compared with 22q11.2 deletion patients with no Parkinson’s disease (22q11.2DS-NPD).

	Rare deleterious coding variant counts^a^											
	22q11.2DS-PD			22q11.2DS-NPD						Analyses		
Subject Identifier	PD1	PD2	PD3	NPD1	NPD2	NPD3	NPD4	NPD5	NPD6	PD Mean (SD)	NPD Mean (SD)	*p*^*b*^
**Genome-wide total**												
Loss-of-function variants	9	7	14	22	17	22	13	9	11	10.0 (3.6)	15.7 (5.6)	0.06
Missense variants	97	98	117	102	79	95	90	82	89	104.0 (11.3)	89.5 (8.4)	0.07
**Candidate gene-sets for 22q11.2DS-PD**												
22q11.2 deletion region genes (46 genes)	1^c^	0	1^c^	0	0	0	0	0	1^c^	0.7 (0.6)	0.2 (0.4)	0.14
Known PD candidate genes (43 genes^d^)	0	0	0^e^	0	0	0	0	0	0	-	-	-
PD-relevant genes (top 1000 candidate genes^f^)												
Loss-of-function variants	1	1	0	1	0	1	1	3	0	0.7 (0.6)	1.7 (1.1)	0.29
Missense variants	12	7	11	3	4	4	5	8	5	10.0 (2.6)	4.8 (1.7)	**0.03**

^a^Rare (MAF<0.01) autosomal heterozygous deleterious variant counts. Only one homozygous variant was identified (missense variant in *ZNF418*, no apparent functional relevance to PD, in PD3)

^b^Nominal *p* value for one-sided independent *t*-test. Non-parametric Wilcoxon testing yielded the same pattern of results

^c^Missense variants in three brain-expressed genes in the proximal typical deletion region, none of which are considered 22q11.2 PD candidate genes [2]: *TRMT2A*, tRNA methyltransferase 2 homolog A [*S*. *cerevisiae*] (PD1); *DGCR2*, DiGeorge syndrome critical region 2 (PD3); *GNB1L*, guanine nucleotide binding protein [G protein], beta-polypeptide 1-like (NPD6)

^d^PD candidate causative and risk genes (S1 Table)

^e^A false positive missense variant in *PARK2* (subject PD3) was not confirmed: Sanger sequencing showed no mutation [2].

^f^Top 1000 genome-wide genes ranked as potential PD-relevant genes using the genome-wide candidate gene prioritization tool, Endeavour

With respect to the gene-set of 43 putative candidate PD genes (S1 Table), no deleterious nonsynonymous variants (Table 2), or copy number or other structural variants, were identified in any of the nine subjects.

Overall the genome-wide burden of rare deleterious missense variants in coding sequence genes was non-significantly greater in the PD cases than in the patients without PD (Table 2). We assessed a candidate gene-set functionally restricted to the top 1000 genome-wide ranked PD-relevant genes, excluding those in the 22q11.2 deletion region or in known PD genes. This revealed a greater burden of rare deleterious missense variants in the 22q11.2DS-PD cases (nominal *p* = 0.03; FDR = 10%; Table 2). The result remained significant (*p* = 0.04) after correcting for the total number of rare deleterious missense variants per subject. To test the specificity of these findings, we assessed between-group differences for burden of rare deleterious missense variants in 10 000 random non-PD relevant gene-sets. We found that <5% of the resulting *p*-values were less than the *p*-value yielded from the top 1000 genome-wide ranked PD-relevant genes (*p* = 0.03), suggesting the results were specific to the PD-relevant gene-set. Results for the few loss-of-function variants were non-significant (Table 2). There were no genome-wide PD-relevant copy number or other structural variants identified [4].

Secondary analyses showed that the genes with these rare missense variants ranked significantly higher in the PD-relevant gene-set in the PD patients (mean rank, 196) than those in the cases without PD (mean rank, 316; *p* = 0.009), and that burden results remained significant using a higher stringency threshold (top 500 ranked candidates in the PD network gene-set, S2 Table; 22q11.2DS-PD mean = 5.7, SD = 2.1 vs. 22q11.2DS-NPD mean = 2.0, SD = 1.7; *p* = 0.03).

Also, a negative control analysis using lifetime history of schizophrenia or seizure(s) in place of PD as the phenotype of interest [4] showed no significant differences in PD network missense variant burden between groups at either stringency threshold (schizophrenia: top 1000 ranked PD-relevant genes, *p* = 0.81; top 500 genes, *p* = 0.54; seizure(s): top 1000 ranked PD-relevant genes, *p* = 0.17; top 500 genes, *p* = 0.77).

Table 3 shows the PD-relevant genes with rare nonsynonymous variants in the subjects with PD. These included *KLF11*, a regulator of monoamine oxidase B expression [37], and *MAP2*, a neuronal cytoskeletal protein found in Lewy bodies in PD patients (Table 3) [38]. There were two genes with variants that affected more than one case with PD (Table 3). The same rare variant (MAF≤0.01) in *LARS2* (rs116826217) was identified in subjects PD2 and PD3. This gene encodes a mitochondrial aminoacyl-tRNA synthetase reported to be significantly down-regulated (~1.45 fold) in substantia nigra dopaminergic neurons of patients with idiopathic PD [39]. Two different rare variants in *TTN* were identified in subjects PD1 and PD3. Although ranked in the top 100 candidates in the PD-relevant gene-set, *TTN* is highly polymorphic and one of the largest genes in the genome, thus caution is warranted in the interpretation of the potential pathogenicity of variants [40].

**Table 3:** Rare nonsynonymous variants in autosomal genes in a genome-wide Parkinson’s Disease-relevant gene-set in three unrelated patients with 22q11.2 deletion-associated Parkinson’s disease.

Case	Gene symbol	Gene Name	Variant type	Rank^a^	Coordinates (GRCh37)		Ref. allele	Alt. allele	Entrez ID	OMIM ID
					**Chr.**	**Position**				
**PD1**	TTN^b^	titin	Missense	57	2	179560789	T	C	7273	188840
	MAP2	microtubule-associated protein 2	Missense	147	2	210543361	C	A	4133	157130
	HAL	histidine ammonia-lyase	Missense	180	12	96371731	C	T	3034	609457
	GPATCH8	G patch domain containing 8	Missense	184	17	42476786	G	A	23131	614396
	DOCK4	dedicator of cytokinesis 4	Missense	213	7	111368481	G	A	9732	607679
	MYH9	myosin, heavy chain 9, non-muscle	Missense	229	22	36681790	G	A	4627	160775
	KHK	ketohexokinase (fructokinase)	Missense	256	2	27320515	G	A	3795	614058
	HSD17B4	hydroxysteroid (17-beta) dehydrogenase 4	Missense	265	5	118844919	C	T	3295	601860
	EYA1	EYA transcriptional coactivator and phosphatase 1	Missense	648	8	72246370	G	A	2138	601653
	ATXN7	ataxin 7	Missense	735	3	63968025	A	T	6314	607640
	RNF123	ring finger protein 123	Missense	804	3	49737107	C	T	63891	614472
	G3BP1	GTPase activating protein (SH3 domain) binding protein 1	Missense	997	5	151176801	G	C	10146	608431
	PDE1A	Phosphodiesterase 1A, calmodulin-dependent	LOF (deletion)	177	2	183106620 to 183106623	GTTT	NA	5136	171890
**PD2**	KLF11	Kruppel-like factor 11	Missense	75	2	10188597	C	T	8462	188840
	GCA	grancalcin, EF-hand calcium binding protein	Missense	86	2	163208877	G	T	25801	176878
	PTPRG	protein tyrosine phosphatase, receptor type, G	Missense	165	3	62189076	C	T	5793	172250
	ADCY6	adenylate cyclase 6	Missense	408	12	49165650	C	T	112	603301
	NFATC1	nuclear factor of activated T-cells, cytoplasmic, calcineurin-dependent 1	Missense	887	18	77171480	T	C	4772	600489
	LARS2^b^	leucyl-tRNA synthetase 2, mitochondrial	Missense	921	3	45537795	G	A	23395	604544
	TYR	tyrosinase	Missense	970	11	89017973	C	T	7299	606933
	C19orf80	chromosome 19 open reading frame 80	LOF (nonsense)	936	19	11350874	C	T	55908	NA
**PD3**	ANKHD1	ankyrin repeat and KH domain containing 1	Missense	22	5	139815809	C	G	54882	610500
	ARG1	arginase 1	Missense	29	6	131904553	C	T	383	608313
	TTN^b^	titin	Missense	57	2	179430433	C	T	7273	188840
	MTMR14	myotubularin related protein 14	Missense	469	3	9714418	A	G	64419	611089
	MC5R	melanocortin 5 receptor	Missense	489	18	13826678	C	T	4161	600042
	DHTKD1	dehydrogenase E1 and transketolase domain containing 1	Missense	502	10	12129639	G	T	55526	614984
	ALDH4A1	aldehyde dehydrogenase 4 family, member A1	Missense	512	1	19209862	A	G	8659	606811
	SMG6	SMG6 nonsense mediated mRNA decay factor	Missense	585	17	2202573	G	A	23293	610963
	HARS	histidyl-tRNA synthetase	Missense	815	5	140070517	C	T	3035	142810
	LARS2^b^	leucyl-tRNA synthetase 2, mitochondrial	Missense	921	3	45537795	G	A	23395	604544
	PGLYRP4	peptidoglycan recognition protein 4	Missense	972	1	153303392	C	T	57115	608198

Abbreviations: Alt. allele, alternate allele; Chr., chromosome; LOF, loss-of-function; NA, not applicable; Ref. allele, reference allele

^a^Genome-wide rank as a potential PD-relevant gene using the genome-wide candidate gene prioritization tool, Endeavour

^b^Variants involving the same gene in two patients with 22q11.2DS-associated Parkinson’s disease (same variant with respect to *LARS2*)

At the most stringent nominal *p*-value threshold of ≤1e^-7^ (corresponding to just 388 SNPs), the mean polygenic risk score was non-significantly greater in the PD cases (-18.6 vs. -23.7; *p* = 0.17; S1 Fig). A similar non-significant trend with more modest relative differences was observed at more lenient SNP nominal p-value thresholds.

## Discussion

This first ever study using WGS in PD provides initial proof-of-principle of the utility of next-generation sequencing for studying the etiology of PD, and the potential advantage of using a genetic model, here, 22q11.2DS. The results provide preliminary evidence that genome-wide burden of rare deleterious variants in genes functionally relevant to PD may collectively act to increase the likelihood of expression of PD in the presence of a 22q11.2 deletion. This is consistent with, and extends to PD, findings using this WGS approach for schizophrenia in 22q11.2DS [4]. These findings will require confirmation in adequately powered larger samples.

The results of this proof-of-principle study suggest that hemizygosity of the 22q11.2 deletion region, together with each individual’s cumulative genome-wide burden of rare deleterious variants in PD-relevant pathways, with perhaps some modification related to cumulative common variants, may form a “multi-hit” pathway to the expression of PD in 22q11.2DS. Collectively, the findings provide an initial glimpse of the potential for WGS to reveal a more complete view of the complex genetic architecture of PD at the individual level that could potentially be generalizable to other forms of PD. Reduced gene dosage in the 22q11.2 region appears to be a more plausible mechanism for increasing risk of early-onset PD in 22q11.2DS than does the unmasking of a recessive allele on the intact 22q11.2 chromosome.

With this sample size, we could not find evidence of a contribution to risk from pathogenic mutations in well-known or other risk genes for PD, nor loss-of-function mutations affecting genes in the broader PD-relevant network. Notably, enrichment of additional rare *ATP13A2* variants was recently reported in *LRRK2-*associated PD [41]. Larger studies of patients with 22q11.2DS-associated PD will help clarify if additional rare variants in known PD genes could impact penetrance of PD in this genetic population. Though non-significant, the finding of a less negative polygenic score in the 22q11.2DS-PD cases suggests the possibility that 22q11.2DS patients who develop PD may have fewer protective PD risk alleles. These preliminary findings provide hypotheses that await testing in larger, well-powered samples that will become feasible as more patients with 22q11.2DS-PD continue to be identified [2, 3, 42–49].

The smaller proximal deletion that occurs in about 10% of cases [50, 51] was identified in one of the 22q11.2DS patients with PD sequenced in this study [2]. Adequately-powered studies will be needed to investigate the molecular nature of the deletion and the potential impact of neighbouring mutations as a possible source of discordance for PD in 22q11.2DS. Notably, there appears to be no relationship between deletion length and expression of other major neuropsychiatric features in 22q11.2DS [52–54]. We found no evidence that nonsynonymous variants near the 22q11.2 deletion breakpoints impacted expression of PD.

The results of this study appear to be consistent with the polygenic genetic architecture expected for a common, complex neurological disorder such as PD. Studies using a comparable design in this and other genetic models of PD (e.g., *LRRK2*-associated PD) could help to reveal generalizable mechanisms relevant to the reduced penetrance associated with most mutations. Non-genetic factors may also be important to include in future designs. For example, Vitamin D deficiency may increase PD risk [55], and is a common finding in 22q11.2DS related to inadequate levels of parathyroid hormone secretion [32, 33, 56]. Prospective studies could help clarify if Vitamin D levels may be involved in mediating PD risk in 22q11.2DS.

The small sample size necessitated analyses focused on a targeted candidate gene-set approach. This included reliance on reported PD-associated genes to seed a network to prioritize rare variants across the genome. More individual PD genes are likely to be discovered, and others may be dropped from such lists as data accrue. Although this initial study produced a nominally significant enrichment of rare putatively damaging missense variants in PD-relevant genes among the PD cases, there was no correction for multiple comparisons. We limited the number of tests to the minimum by considering only gene-sets with relevance to PD. These findings will require replication in adequately powered samples. We were underpowered in this preliminary study to include clinical covariates, including other neuropsychiatric phenotypes, in our analyses. The clinical phenotype of 22q11.2DS patients with PD reported to date appears consistent with the variable expression and reduced penetrance of the associated features that is characteristic of 22q11.2DS [2, 3, 42–49]. However, there were no significant differences in the PD network variant burden using other major neurological phenotypes (e.g., expression of schizophrenia or seizures) as the grouping variable, suggesting the findings may be specific to PD-relevant genes.

Advances in WGS bioinformatics methods will permit informative analyses of non-coding regions in future studies. The 22q11.2 region includes *DGCR8*, a key gene in the biogenesis of brain microRNAs, in addition to seven microRNAs [57]. It remains possible that one or more of the cases without PD may go on to develop PD at a later age, limiting the power to determine between-group differences. Evaluating clinical and neuroimaging phenotypes in genetic studies of patients with 22q11.2DS without a diagnosis of PD may help clarify disease risk. We recently found that adults with 22q11.2DS without a diagnosis of PD exhibit olfactory and motor deficits compared with age-matched healthy controls [58]. The true penetrance of PD in adults with 22q11.2DS remains to be reliably estimated, and there are as yet no predictive clinical markers of PD. Longitudinal studies will help resolve such questions. For the six cases without PD, all but one (NPD1) had reached or was beyond the reported age-at-onset range for 22q11.2DS-associated early-onset PD [2, 3, 42–46]. The absence of PD pathology was confirmed in one case where brain tissue was available (NPD4) [2], and there was no evidence of nigrostriatal dopamine loss in two others (NPD1 and NPD6) on PET neuroimaging using ^11^C-dihydrotetrabenazine relative to controls [58, 59].

The results of this study represent an important first step in appreciating how WGS can help to elucidate the genetic etiology of early-onset PD in 22q11.2DS. These findings may have implications for other genetic models of PD and for idiopathic PD. Eventually such molecular results could help to inform early identification and intervention strategies for individuals at risk.

## Supporting information

S1 Fig Distribution boxplots of polygenic risk scores for patients with 22q11.2 deletion syndrome and Parkinson’s disease (22q11.2DS-PD) and those without Parkinson’s disease (22q11.2DS-NPD). The mean polygenic score (open diamond) is non-significantly greater in the 22q11.2DS-PD group than the 22q11.2DS-NPD group (*p* = 0.17) at the most stringent *p*-value threshold (left panel, (≤1e^-7^). This pattern was not observed at lower *p-*value thresholds (e.g., ≤1e^-3^) shown here in the right panel for comparison purposes). See Methods for details.
(DOCX)

S1 Table List of 43 putative candidate genes implicated in Parkinson’s disease. (DOCX)

S2 Table List of genes in Parkinson’s disease-relevant gene-sets. (XLSX)

S3 Table 22q11.2 deletion breakpoints of individuals with 22q11.2 deletion syndrome with whole-genome sequencing results. (DOCX)

## References

[1] Klein C, Westenberger A. Genetics of Parkinson's disease. Cold Spring Harb Perspect Med. 2012;2: a008888 doi: 10.1101/cshperspect.a008888 22315721 10.1101/cshperspect.a008888 PMC3253033

[2] Butcher N, Kiehl T, Hazrati L, Chow E, Rogaeva E, Lang A, et al Association between early-onset Parkinson disease and 22q11.2 deletion syndrome: Identification of a novel genetic form of Parkinson disease and its clinical implications. JAMA Neurology. 2013;70: 1359–1366. doi: 10.1001/jamaneurol.2013.3646 24018986 10.1001/jamaneurol.2013.3646 PMC4464823

[3] Mok KY, Sheerin U, Simon-Sanchez J, Salaka A, Chester L, Escott-Price V, et al Deletions at 22q11.2 in idiopathic Parkinson's disease: a combined analysis of genome-wide association data. Lancet Neurol. 2016;15: 585–596. doi: 10.1016/S1474-4422(16)00071-5 27017469 10.1016/S1474-4422(16)00071-5 PMC4828586

[4] Merico D, Zarrei M, Costain G, Ogura L, Alipanahi B, Gazzellone MJ, et al Whole-genome sequencing suggests schizophrenia risk mechanisms in humans with 22q11.2 deletion syndrome. G3 (Bethesda). 2015;5: 2453–2461. 26384369 10.1534/g3.115.021345 PMC4632064

[5] Bassett AS, Chow EW, AbdelMalik P, Gheorghiu M, Husted J, Weksberg R. The schizophrenia phenotype in 22q11 deletion syndrome. Am J Psychiatry. 2003;160: 1580–1586. doi: 10.1176/appi.ajp.160.9.1580 12944331 10.1176/appi.ajp.160.9.1580 PMC3276594

[6] Bassett AS, Marshall CR, Lionel AC, Chow EW, Scherer SW. Copy number variations and risk for schizophrenia in 22q11.2 deletion syndrome. Hum Mol Genet. 2008;17: 4045–4053. doi: 10.1093/hmg/ddn307 18806272 10.1093/hmg/ddn307 PMC2638574

[7] Butcher N, Chow E, Costain G, Karas D, Ho A, Bassett A. Functional outcomes of adults with 22q11.2 deletion syndrome. Genet Med. 2012;14: 836–843. doi: 10.1038/gim.2012.66 22744446 10.1038/gim.2012.66 PMC3465579

[8] Van L, Butcher NJ, Costain G, Ogura L, Chow EW, Bassett AS. Fetal growth and gestational factors as predictors of schizophrenia in 22q11.2 deletion syndrome. Genet Med. 2016;18: 350–355. doi: 10.1038/gim.2015.84 26087175 10.1038/gim.2015.84

[9] Yuen RK, Thiruvahindrapuram B, Merico D, Walker S, Tammimies K, Hoang N, et al Whole-genome sequencing of quartet families with autism spectrum disorder. Nat Med. 2015;21: 185–191. doi: 10.1038/nm.3792 25621899 10.1038/nm.3792

[10] Kiehl TR, Chow EWC, Mikulis DJ, George SR, Bassett AS. Neuropathologic features in adults with 22q11.2 deletion syndrome. Cereb Cortex. 2009;19: 153–164. doi: 10.1093/cercor/bhn066 18483005 10.1093/cercor/bhn066

[11] Wang K, Li M, Hakonarson H. ANNOVAR: functional annotation of genetic variants from high-throughput sequencing data. Nucleic Acids Res. 2010;38: e164 doi: 10.1093/nar/gkq603 20601685 10.1093/nar/gkq603 PMC2938201

[12] Genomes Project C, Auton A, Brooks LD, Durbin RM, Garrison EP, Kang HM, et al A global reference for human genetic variation. Nature. 2015;526: 68–74. doi: 10.1038/nature15393 26432245 10.1038/nature15393 PMC4750478

[13] Tennessen JA, Bigham AW, O'Connor TD, Fu W, Kenny EE, Gravel S, et al Evolution and functional impact of rare coding variation from deep sequencing of human exomes. Science. 2012;337: 64–69. doi: 10.1126/science.1219240 22604720 10.1126/science.1219240 PMC3708544

[14] Lek M, Karczewski KJ, Minikel EV, Samocha KE, Banks E, Fennell T, et al Analysis of protein-coding genetic variation in 60,706 humans. Nature. 2016;536: 285–291. doi: 10.1038/nature19057 27535533 10.1038/nature19057 PMC5018207

[15] Pollard KS, Hubisz MJ, Rosenbloom KR, Siepel A. Detection of nonneutral substitution rates on mammalian phylogenies. Genome Res. 2010;20: 110–121. doi: 10.1101/gr.097857.109 19858363 10.1101/gr.097857.109 PMC2798823

[16] Ng PC, Henikoff S. Predicting deleterious amino acid substitutions. Genome Res. 2001;11: 863–874. doi: 10.1101/gr.176601 11337480 10.1101/gr.176601 PMC311071

[17] Adzhubei IA, Schmidt S, Peshkin L, Ramensky VE, Gerasimova A, Bork P, et al A method and server for predicting damaging missense mutations. Nat Methods. 2010;7: 248–249. doi: 10.1038/nmeth0410-248 20354512 10.1038/nmeth0410-248 PMC2855889

[18] Reva B, Antipin Y, Sander C. Predicting the functional impact of protein mutations: application to cancer genomics. Nucleic Acids Res. 2011;39: e118 doi: 10.1093/nar/gkr407 21727090 10.1093/nar/gkr407 PMC3177186

[19] Kircher M, Witten DM, Jain P, O'Roak BJ, Cooper GM, Shendure J. A general framework for estimating the relative pathogenicity of human genetic variants. Nat Genet. 2014;46: 310–315. doi: 10.1038/ng.2892 24487276 10.1038/ng.2892 PMC3992975

[20] Schwarz JM, Cooper DN, Schuelke M, Seelow D. MutationTaster2: mutation prediction for the deep-sequencing age. Nat Methods. 2014;11: 361–362. doi: 10.1038/nmeth.2890 24681721 10.1038/nmeth.2890

[21] Ogaki K, Ross OA. Chromosome 22q11.2 deletion may contain a locus for recessive early-onset Parkinson's disease. Parkinsonism Relat Disord. 2014;20: 945–946. doi: 10.1016/j.parkreldis.2014.06.020 25001314 10.1016/j.parkreldis.2014.06.020 PMC4143462

[22] Guna A, Butcher NJ, Bassett AS. Comparative mapping of the 22q11.2 deletion region and the potential of simple model organisms. J Neurodev Disord. 2015;7: 18 doi: 10.1186/s11689-015-9113-x 26137170 10.1186/s11689-015-9113-x PMC4487986

[23] Nalls MA, Pankratz N, Lill CM, Do CB, Hernandez DG, Saad M, et al Large-scale meta-analysis of genome-wide association data identifies six new risk loci for Parkinson's disease. Nat Genet. 2014;46: 989–993. doi: 10.1038/ng.3043 25064009 10.1038/ng.3043 PMC4146673

[24] Lill CM, Roehr JT, McQueen MB, Kavvoura FK, Bagade S, Schjeide BM, et al Comprehensive research synopsis and systematic meta-analyses in Parkinson's disease genetics: The PDGene database. PLoS Genet. 2012;8: e1002548 doi: 10.1371/journal.pgen.1002548 22438815 10.1371/journal.pgen.1002548 PMC3305333

[25] Spataro N, Calafell F, Cervera-Carles L, Casals F, Pagonabarraga J, Pascual-Sedano B, et al Mendelian genes for Parkinson's disease contribute to the sporadic forms of the disease. Hum Mol Genet. 2015;24: 2023–2034. doi: 10.1093/hmg/ddu616 25504046 10.1093/hmg/ddu616

[26] Aerts S, Lambrechts D, Maity S, Van Loo P, Coessens B, De Smet F, et al Gene prioritization through genomic data fusion. Nat Biotechnol. 2006;24: 537–544. doi: 10.1038/nbt1203 16680138 10.1038/nbt1203

[27] Tranchevent LC, Ardeshirdavani A, ElShal S, Alcaide D, Aerts J, Auboeuf D, et al Candidate gene prioritization with Endeavour. Nucleic Acids Res. 2016;44: W117–121. doi: 10.1093/nar/gkw365 27131783 10.1093/nar/gkw365 PMC4987917

[28] Tranchevent LC, Barriot R, Yu S, Van Vooren S, Van Loo P, Coessens B, et al ENDEAVOUR update: a web resource for gene prioritization in multiple species. Nucleic Acids Res. 2008;36: W377–384. doi: 10.1093/nar/gkn325 18508807 10.1093/nar/gkn325 PMC2447805

[29] Son CG, Bilke S, Davis S, Greer BT, Wei JS, Whiteford CC, et al Database of mRNA gene expression profiles of multiple human organs. Genome Res. 2005;15: 443–450. doi: 10.1101/gr.3124505 15741514 10.1101/gr.3124505 PMC551571

[30] Gene Ontology C, Blake JA, Dolan M, Drabkin H, Hill DP, Li N, et al Gene Ontology annotations and resources. Nucleic Acids Res. 2013;41: D530–535. doi: 10.1093/nar/gks1050 23161678 10.1093/nar/gks1050 PMC3531070

[31] International Schizophrenia C, Purcell SM, Wray NR, Stone JL, Visscher PM, O'Donovan MC, et al Common polygenic variation contributes to risk of schizophrenia and bipolar disorder. Nature. 2009;460: 748–752. doi: 10.1038/nature08185 19571811 10.1038/nature08185 PMC3912837

[32] Bassett AS, McDonald-McGinn DM, Devriendt K, Digilio MC, Goldenberg P, Habel A, et al Practical guidelines for managing patients with 22q11.2 deletion syndrome. J Pediatr. 2011;159: 332–339 doi: 10.1016/j.jpeds.2011.02.039 21570089 10.1016/j.jpeds.2011.02.039 PMC3197829

[33] Fung WL, Butcher NJ, Costain G, Andrade DM, Boot E, Chow EW, et al Practical guidelines for managing adults with 22q11.2 deletion syndrome. Genet Med. 2015;17: 599–609. doi: 10.1038/gim.2014.175 25569435 10.1038/gim.2014.175 PMC4526275

[34] McDonald-McGinn DM, Sullivan KE, Marino B, Philip N, Swillen A, Vorstman JAS, et al 22q11.2 deletion syndrome. Nature Reviews Disease Primers. 2015;1: 15071 doi: 10.1038/nrdp.2015.71 27189754 10.1038/nrdp.2015.71 PMC4900471

[35] Hicks DG, Janarthanan BR, Vardarajan R, Kulkarni SA, Khoury T, Dim D, et al The expression of TRMT2A, a novel cell cycle regulated protein, identifies a subset of breast cancer patients with HER2 over-expression that are at an increased risk of recurrence. BMC Cancer. 2010;10: 108 doi: 10.1186/1471-2407-10-108 20307320 10.1186/1471-2407-10-108 PMC2859753

[36] Kajiwara K, Nagasawa H, Shimizu-Nishikawa K, Ookura T, Kimura M, Sugaya E. Cloning of SEZ-12 encoding seizure-related and membrane-bound adhesion protein. Biochem Biophys Res Commun. 1996;222: 144–148. doi: 10.1006/bbrc.1996.0712 8630060 10.1006/bbrc.1996.0712

[37] Ou XM, Chen K, Shih JC. Dual functions of transcription factors, transforming growth factor-beta-inducible early gene (TIEG)2 and Sp3, are mediated by CACCC element and Sp1 sites of human monoamine oxidase (MAO) B gene. J Biol Chem. 2004;279: 21021–21028. doi: 10.1074/jbc.M312638200 15024015 10.1074/jbc.M312638200

[38] D'Andrea MR, Ilyin S, Plata-Salaman CR. Abnormal patterns of microtubule-associated protein-2 (MAP-2) immunolabeling in neuronal nuclei and Lewy bodies in Parkinson's disease substantia nigra brain tissues. Neurosci Lett. 2001;306: 137–140. 11406314 10.1016/s0304-3940(01)01811-0

[39] Simunovic F, Yi M, Wang Y, Macey L, Brown LT, Krichevsky AM, et al Gene expression profiling of substantia nigra dopamine neurons: further insights into Parkinson's disease pathology. Brain. 2009;132: 1795–1809. doi: 10.1093/brain/awn323 19052140 10.1093/brain/awn323 PMC2724914

[40] Chauveau C, Rowell J, Ferreiro A. A rising titan: TTN review and mutation update. Hum Mutat. 2014;35: 1046–1059. doi: 10.1002/humu.22611 24980681 10.1002/humu.22611

[41] Lubbe SJ, Escott-Price V, Gibbs JR, Nalls MA, Bras J, Price TR, et al Additional rare variant analysis in Parkinson's disease cases with and without known pathogenic mutations: evidence for oligogenic inheritance. Hum Mol Genet. 2016. 10.1093/hmg/ddw348 PMC5418836 27798102

[42] Rehman AF, Dhamija R, Williams ES, Barrett MJ. 22q11.2 deletion syndrome presenting with early-onset Parkinson's disease. Mov Disord. 2015;30: 1289–1290. doi: 10.1002/mds.26305 26195290 10.1002/mds.26305

[43] Verhoeven WM, Egger JI. Atypical antipsychotics and relapsing psychoses in 22q11.2 deletion syndrome: A long-term evaluation of 28 patients. Pharmacopsychiatry. 2015;Epub: Feb 5th 2015. 10.1055/s-0034-1398612 25654302

[44] Zaleski C, Bassett AS, Tam K, Shugar AL, Chow EW, McPherson E. The co-occurrence of early onset Parkinson disease and 22q11.2 deletion syndrome. Am J Med Genet A. 2009;149A: 525–528. doi: 10.1002/ajmg.a.32650 19208384 10.1002/ajmg.a.32650 PMC3295831

[45] Booij J, van Amelsvoort T, Boot E. Co-occurrence of early-onset Parkinson disease and 22q11.2 deletion syndrome: Potential role for dopamine transporter imaging. Am J Med Genet A. 2010;152A: 2937–2938. doi: 10.1002/ajmg.a.33665 20949509 10.1002/ajmg.a.33665

[46] Oki M, Hori S, Asayama S, Wate R, Kaneko S, Kusaka H. Early-onset Parkinson's disease associated with chromosome 22q11.2 deletion syndrome. Intern Med. 2016;55: 303–305. doi: 10.2169/internalmedicine.55.5485 26831029 10.2169/internalmedicine.55.5485

[47] Foo JN, Lee J, Tan LC, Liu J, Tan EK. Large 3-Mb deletions at 22q11.2 locus in Parkinson's disease and schizophrenia. Mov Disord. 2016;31: 1924–1925. doi: 10.1002/mds.26822 27787935 10.1002/mds.26822

[48] Pollard R, Hannan M, Tanabe J, Berman BD. Early-onset Parkinson disease leading to diagnosis of 22q11.2 deletion syndrome. Parkinsonism Relat Disord. 2016;25: 110–111. doi: 10.1016/j.parkreldis.2016.01.027 26868161 10.1016/j.parkreldis.2016.01.027

[49] Vogels A, Verhoeven WM, Tuinier S, DeVriendt K, Swillen A, Curfs LM, et al The psychopathological phenotype of velo-cardio-facial syndrome. Ann Genet. 2002;45: 89–95. 12119217 10.1016/s0003-3995(02)01114-0

[50] Edelmann L, Pandita RK, Morrow BE. Low-copy repeats mediate the common 3-Mb deletion in patients with velo-cardio-facial syndrome. Am J Hum Genet. 1999;64: 1076–1086. 10090893 10.1086/302343 PMC1377832

[51] Shaikh TH, Kurahashi H, Saitta SC, O'Hare AM, Hu P, Roe BA, et al Chromosome 22-specific low copy repeats and the 22q11.2 deletion syndrome: genomic organization and deletion endpoint analysis. Hum Mol Genet. 2000;9: 489–501. 10699172 10.1093/hmg/9.4.489

[52] Gothelf D, Schaer M, Eliez S. Genes, brain development and psychiatric phenotypes in velo-cardio-facial syndrome. Develop Disabil Res Rev. 2008;14: 59–68. 10.1002/ddrr.9 18636637

[53] Weksberg R, Stachon AC, Squire JA, Moldovan L, Bayani J, Meyn S, et al Molecular characterization of deletion breakpoints in adults with 22q11 deletion syndrome. Hum Genet. 2007;120: 837–845. doi: 10.1007/s00439-006-0242-x 17028864 10.1007/s00439-006-0242-x PMC3139629

[54] Karayiorgou M, Simon TJ, Gogos JA. 22q11.2 microdeletions: linking DNA structural variation to brain dysfunction and schizophrenia. Nat Rev Neurosci. 2010;11: 402–416. doi: 10.1038/nrn2841 20485365 10.1038/nrn2841 PMC2977984

[55] Wang L, Evatt ML, Maldonado LG, Perry WR, Ritchie JC, Beecham GW, et al Vitamin D from different sources is inversely associated with Parkinson disease. Mov Disord. 2015;30: 560–566. doi: 10.1002/mds.26117 25545356 10.1002/mds.26117 PMC4390412

[56] Kelley L, Sanders AF, Beaton EA. Vitamin D deficiency, behavioral atypicality, anxiety and depression in children with chromosome 22q11.2 deletion syndrome. J Dev Orig Health Dis. 2016;7: 616–625. doi: 10.1017/S2040174416000428 27827293 10.1017/S2040174416000428 PMC5922262

[57] Merico D, Costain G, Butcher NJ, Warnica W, Ogura L, Alfred SE, et al MicroRNA dysregulation, gene networks, and risk for schizophrenia in 22q11.2 deletion syndrome. Front Neurol. 2014;5: 238 doi: 10.3389/fneur.2014.00238 25484875 10.3389/fneur.2014.00238 PMC4240070

[58] Butcher N, Marras C, Pondal M, Rusjan P, Boot E, Christopher L, et al. Neuroimaging and clinical features in adults with a 22q11.2 deletion at risk of Parkinson's disease Brain. 2017; Forthcoming. 10.1093/brain/awx053 28369257

[59] Butcher N, Marras C, Pondal M, Rusjan P, Christopher L, Strafella A, et al Investigating prodromal markers of Parkinson’s disease in adults with hemizygous 22q11.2 deletions. Mov Disord. 2015;30 (S1): S1035.

