# Supplementary material for: Whole-genome sequencing suggests mechanisms for 22q11.2 deletion-associated Parkinson’s disease
Source: PLoS One. 2017 Apr 21;12(4):e0173944. doi: 10.1371/journal.pone.0173944 (PMC5400231; doi:10.1371/journal.pone.0173944)
Supplement: S1 Fig — The mean polygenic score (open diamond) is non-significantly greater in the 22q11.2DS-PD group than the 22q11.2DS-NPD group (p = 0.17) at the most stringent p-value threshold (left panel, (≤1e-7). This pattern was not observed at lower p-value thresholds (e.g., ≤1e-3) shown here in the right panel for comparison purposes). See Methods for details. (DOCX) [file pone.0173944.s001.docx]

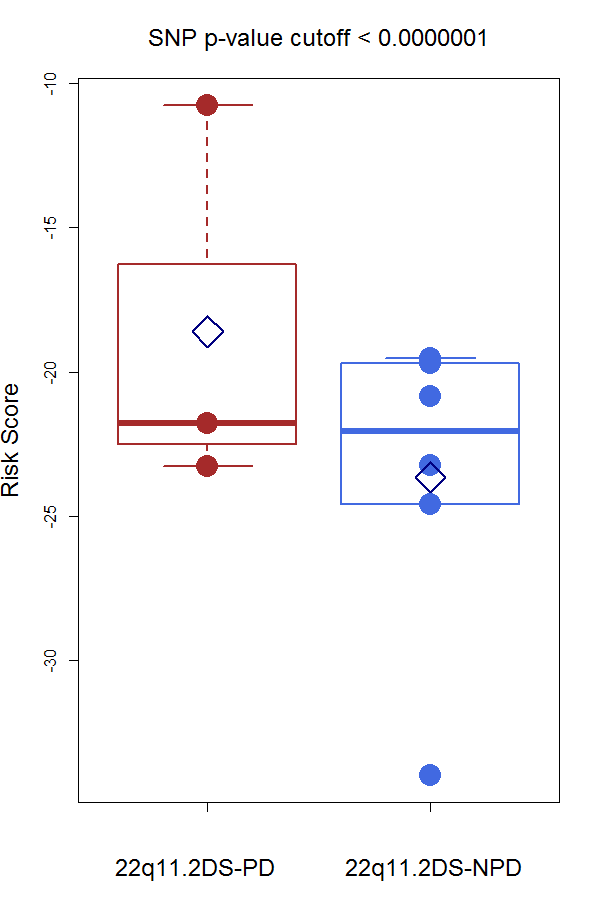

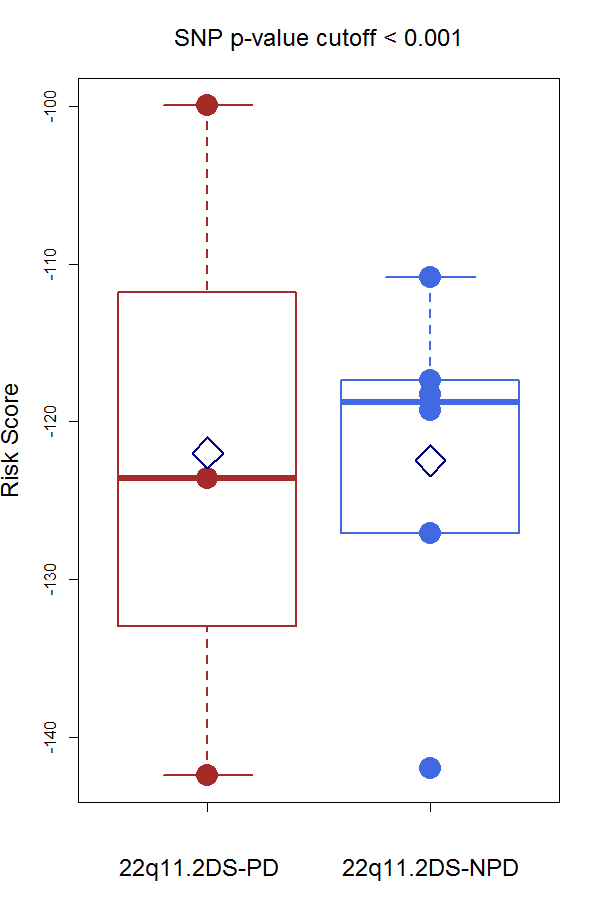


**S1 Fig. Distribution Boxplots of Polygenic Risk Scores for Patients with 22q11.2 Deletion Syndrome and Parkinson’s Disease (22q11.2DS-PD) And Those Without Parkinson’s Disease (22q11.2DS-NPD).** The mean polygenic score (open diamond) is non-significantly greater in the 22q11.2DS-PD group than the 22q11.2DS-NPD group (*p*=0.17) at the most stringent *p*-value threshold (left panel, (≤1e^-7^). This pattern was not observed at lower *p-*value thresholds (e.g., ≤1e^-3^) shown here in the right panel for comparison purposes). See Methods for details.
