# Supplementary material for: Whole-genome sequencing suggests mechanisms for 22q11.2 deletion-associated Parkinson’s disease
Source: PLoS One. 2017 Apr 21;12(4):e0173944. doi: 10.1371/journal.pone.0173944 (PMC5400231; doi:10.1371/journal.pone.0173944)
Supplement: S1 Table — (DOCX) [file pone.0173944.s002.docx]

S1 Table. List of 43 Putative Candidate Genes Implicated in Parkinson’s Disease.

|  | |  | | |  |
| --- | --- | --- | --- | --- | --- |
| **Gene name (n=43^a^)** | **Type^b^** | | **Remarks^c^** |  | |
| LRRK2 (PARK8) | Mendelian D / Risk SNP | | Confirmed / PDgene GWAS top hit |  |  |
| SNCA (PARK1/4) | Mendelian D / Risk SNP | | Confirmed / PDgene GWAS top hit |  |  |
| VPS35 (PARK17) | Mendelian D | | Confirmed |  |  |
| EIF4G1 (PARK18) | Mendelian D | | Unconfirmed |  |  |
| GIGYF2 (PARK11) | Mendelian D | | Unconfirmed |  |  |
| HTRA2 (PARK13) | Mendelian D | | Unconfirmed |  |  |
| UCHL1 (PARK5) | Mendelian D | | Unconfirmed (single family) |  |  |
| ATP13A2 (PARK9) | Mendelian R | | Confirmed |  |  |
| DJ-1 (PARK7) | Mendelian R | | Confirmed |  |  |
| FBXO7 (PARK15) | Mendelian R | | Confirmed |  |  |
| PARK2 (PARK2) | Mendelian R | | Confirmed |  |  |
| PINK1 (PARK6) | Mendelian R | | Confirmed |  |  |
| PLA2G6 (PARK14) | Mendelian R | | Confirmed |  |  |
| DNAJC6 (PARK19) | Mendelian R | | Recently identified (2012) |  |  |
| SYNJ1 (PARK20) | Mendelian R | | Recently identified (2013) |  |  |
| GBA | Complex | | Confirmed |  |  |
| ADH1C | Complex | | Unconfirmed |  |  |
| MC1R | Complex | | Unconfirmed |  |  |
| TH | Complex | | Unconfirmed (single patient) |  |  |
| GCH1 | Complex / Risk SNP | | Recently identified (2014) / PDgene GWAS top hit |  |  |
| SMPD1 | Complex | | Recently identified (2013) |  |  |
| ATXN2 | Complex | | Usually cause SCA2^d^ |  |  |
| ATXN3 | Complex | | Usually cause SCA3^d^ |  |  |
| TBP | Complex | | Usually cause SCA17^d^ |  |  |
| ASH1L | Risk SNP | | PDgene GWAS top hit |  |  |
| BCKDK | Risk SNP | | PDgene GWAS top hit |  |  |
| BST1 | Risk SNP | | PDgene GWAS top hit |  |  |
| CCDC62 | Risk SNP | | PDgene GWAS top hit |  |  |
| DLG2 | Risk SNP | | PDgene GWAS top hit |  |  |
| FAM47E | Risk SNP | | PDgene GWAS top hit |  |  |
| GPNMB | Risk SNP | | PDgene GWAS top hit |  |  |
| HLA-DQB1 | Risk SNP | | PDgene GWAS top hit |  |  |
| INPP5F | Risk SNP | | PDgene GWAS top hit |  |  |
| MAPT | Risk SNP | | PDgene GWAS top hit |  |  |
| MCCC1 | Risk SNP | | PDgene GWAS top hit |  |  |
| NUCKS1 | Risk SNP | | PDgene GWAS top hit |  |  |
| RIT2 | Risk SNP | | PDgene GWAS top hit |  |  |
| SIPA1L2 | Risk SNP | | PDgene GWAS top hit |  |  |
| STK39 | Risk SNP | | PDgene GWAS top hit |  |  |
| TMEM175 | Risk SNP | | PDgene GWAS top hit |  |  |
| TMEM229B | Risk SNP | | PDgene GWAS top hit |  |  |
| TMPRSS9 | Risk SNP | | PDgene GWAS top hit |  |  |
| UBOX5 | Risk SNP | | PDgene GWAS top hit |  |  |

^a^Genes include those described in the scientific literature and/or, OMIM entries for Parkinson’s disease (#168600), and from the PDgene database of genome-wide association study (GWAS) common variant findings ([www.pdgene.org](http://www.pdgene.org)). The latter includes the most significant hits thought to be associated with protein-coding genes (single-nucleotide polymorphisms; SNPs) generated from up to 15 independent genome-wide association study datasets of European descent. Details on the assessed datasets as well as genotyping procedures and statistical analyses can be found in Nalls et al. 2014 and online at [www.pdgene.org](http://www.pdgene.org).

^b^Denotes primary type of reported involvement in Parkinson’s disease. Note that most genes with variants involved in PD are associated with reduced penetrance that may vary with age, ethnicity, and other factors. “Mendelian” refers to genes associated with familial forms of Parkinson’s disease with Mendelian dominant (Mendelian D) or recessive (Mendelian R) patterns of inheritance. “Complex” includes genes associated with Parkinson’s disease risk but not known to cause familial Parkinson’s disease (i.e., risk variants with strongly reduced penetrance). GWAS “Risk SNPs” includes genes associated with well-replicated though common low risk susceptibility loci.

^c^We opted to assess all genes (e.g., confirmed, unconfirmed, and rarely) reported to be involved in Parkinson’s disease in order to limit the possibility of a false negative with respect to susceptibility to Parkinson’s disease in patients with 22q11.2DS.

^d^Trinucleotide repeat expansions in genes *ATXN2, ATXN3*, and *TBP* are more typically associated with the spinocerebellar ataxias (SCA2, SCA3, SCA17, respectively) but may also be rare causes of a late-onset Parkinson’s disease phenotype*.*
