# Supplementary material for: Whole-genome sequencing suggests mechanisms for 22q11.2 deletion-associated Parkinson’s disease
Source: PLoS One. 2017 Apr 21;12(4):e0173944. doi: 10.1371/journal.pone.0173944 (PMC5400231; doi:10.1371/journal.pone.0173944)
Supplement: S3 Table — (DOCX) [file pone.0173944.s004.docx]

S3 Table. 22q11.2 Deletion Breakpoints of Individuals with 22q11.2 Deletion Syndrome with Whole-genome Sequencing Results

|  | |  | | |  |
| --- | --- | --- | --- | --- | --- |
| **Subject identifier** | **Deletion size (bp)** | | **Breakpoints Coordinates (bp)**^a^ |  | |
| PD1 | 1,422,000 | | 18,890,000 (-232,000) to 20,312,000 (+404,000) |  |  |
| PD2 | 2,574,000 | | 18,890,000 (-232,000) to 21,464,000 (+334,000) |  |  |
| PD3 | 2,574,000 | | 18,890,000 (-232,000) to 21,464,000 (+334,000) |  |  |
| NPD1 | 2,574,000 | | 18,890,000 (-232,000) to 21,464,000 (+334,000) |  |  |
| NPD2 | 2,574,000 | | 18,890,000 (-232,000) to 21,464,000 (+334,000) |  |  |
| NPD3 | 2,574,000 | | 18,890,000 (-232,000) to 21,464,000 (+334,000) |  |  |
| NPD4 | 2,574,000 | | 18,890,000 (-232,000) to 21,464,000 (+334,000) |  |  |
| NPD5 | 2,574,000 | | 18,890,000 (-232,000) to 21,464,000 (+334,000) |  |  |
| NPD6 | 2,922,000 | | 18,890,000 (-232,000) to 21,812,000 (+106,000) |  |  |

Abbreviations: bp, base pairs. PD1 to PD3 indicates subjects with 22q11.2 deletion syndrome (22q11.2DS) with Parkinson’s disease. NPD1 to NPD6 indicates subjects with 22q11.2DS without a diagnosis of Parkinson’s disease

^a^Numbers in brackets indicate possible variability of the true breakpoints due to the termination of the 22q11.2 deletion copy number variant within segmental duplications
